# Lactate dehydrogenase to albumin ratio is associated with in-hospital mortality in patients with acute heart failure: Data from the MIMIC-III database

**DOI:** 10.1515/med-2024-0901

**Published:** 2024-03-19

**Authors:** Xiangjun Xia, Suisai Tan, Runhong Zeng, Can Ouyang, Xiabin Huang

**Affiliations:** Department of Cardiology, Yiyang Central Hospital, Yiyang, 410215, Hunan, China; Hunan Province Clinical Medical Technology Demonstration Base for Complex Coronary Lesions, Yiyang, Hunan, China; Department of Vascular Surgery, Yiyang Central Hospital, Yiyang, 410215, Hunan, China; The Traditional Chinese Medical Hospital of Xiangtan County, Xiangtan, Hunan, China

**Keywords:** acute heart failure, lactate dehydrogenase to albumin ratio, in-hospital mortality, MIMIC-III, data mining

## Abstract

The effect of the lactate dehydrogenase to albumin ratio (LAR) on the survival of patients with acute heart failure (AHF) is unclear. We aimed to analyze the impact of LAR on survival in patients with AHF. We retrieved eligible patients for our study from the Monitoring in Intensive Care Database III. For each patient in our study, we gathered clinical data and demographic information. We conducted multivariate logistic regression modeling and smooth curve fitting to assess whether the LAR score could be used as an independent indicator for predicting the prognosis of AHF patients. A total of 2,177 patients were extracted from the database. Survivors had an average age of 69.88, whereas nonsurvivors had an average age of 71.95. The survivor group had a mean LAR ratio of 13.44, and the nonsurvivor group had a value of 17.38. LAR and in-hospital mortality had a nearly linear correlation, according to smooth curve fitting (*P* < 0.001). According to multivariate logistic regression, the LAR may be an independent risk factor in predicting the prognosis of patients with AHF (odd ratio = 1.09; *P* < 0.001). The LAR ratio is an independent risk factor associated with increased in-hospital mortality rates in patients with AHF.

## 1 Introduction

Heart failure (HF) is a chronic and progressive syndrome that afflicts millions of individuals worldwide and remains a leading cause of morbidity and mortality [1]. Acute heart failure (AHF), in particular, represents a medical emergency that necessitates prompt and aggressive intervention [2]. Despite advances in the management of HF, the prognosis of AHF remains unsatisfactory, with high rates of hospital readmission and mortality [3]. As such, there is an urgent need for new biomarkers that can enhance risk stratification and guide therapeutic decision-making in AHF.

The lactate dehydrogenase (LDH) to albumin ratio (LAR), expressed as LDH/albumin, is a recently proposed biomarker that shows promise in predicting outcomes in various disease states, including cancer, liver disease, and sepsis [4,5]. The LAR represents the ratio of LDH to albumin levels in the blood and has been suggested as a marker of systemic inflammation and oxidative stress [6,7]. However, the clinical usefulness of LAR in predicting outcomes in AHF patients remains uncertain, and further research is needed to validate its efficacy in this population.

This study aims to investigate the relationship between left atrial remodeling (LAR) and AHF and evaluate its prognostic value in this specific population. Our research expands on previous studies by examining the potential of LAR as a prognostic biomarker in a sizable group of AHF patients. The results of this study could significantly impact the management of AHF and the development of innovative therapeutic approaches.

## 2 Materials and methods

### 2.1 Data source

Medical Information Mart for Intensive Care (MIMIC-III) is a publicly available critical care database designed to support research and development of intelligent systems in healthcare. It is one of the largest and most comprehensive electronic health record databases available, containing detailed clinical data from over 50,000 patients who received care in the intensive care units (ICUs) at Beth Israel Deaconess Medical Center between 2001 and 2012 [8,9]. The database is freely available to researchers, clinicians, and developers around the world who are working to improve healthcare through artificial intelligence and machine learning. It is maintained by the Laboratory for Computational Physiology at the Massachusetts Institute of Technology and has been used in thousands of research studies and projects since its inception [10,11]. MIMIC-III includes de-identified data such as demographic information, vital signs, laboratory results, medications, diagnoses, procedures, and notes from clinicians. The data are organized into tables using a relational database model, making it easy to query and analyze using standard SQL commands [10]. The present study adhered strictly to the Strengthening the Reporting of Observational Studies in Epidemiology guidelines to ensure transparency and accuracy of our reporting. In addition, the study was carried out in full compliance with the Declaration of Helsinki (2013 revision), which sets forth ethical principles for medical research involving human subjects [12,13].


**Ethics statement:** The patients/participants provided their written informed consent to participate in this study.

### 2.2 Study population

Our study focused on patients with HF for the first time during their admission to the ICU. The diagnosis of AHF was made based on the International Classification of Diseases, Ninth and Tenth Revisions. To be eligible for inclusion in our study, patients had to meet the following criteria: (1) 18 years of age or older, (2) admitted to the ICU for a minimum of 24 h, and (3) had only one ICU admission. Conversely, patients were excluded if they met any of the following criteria: (1) were under 18 years of age, (2) had incomplete LDH or albumin data, or (3) were in the ICU for less than 24 h.

### 2.3 Variables

All data were extracted within 24 h of the patient’s admission to the ICU. The variables extracted included demographic characteristics, vital signs, interventions, comorbidities, laboratory indicators, and scoring systems. Demographic characteristics included age, sex, and race. Vital signs measured on admission for the first time were recorded, such as heart rate (HR), respiratory rate, systolic blood pressure (SBP), and diastolic blood pressure (DBP). Interventions, such as vasopressin, were recorded within 24 h of admission. Laboratory parameters, such as hemoglobin, white blood cell (WBC), platelet count, blood glucose, sodium, blood urea nitrogen (BUN), creatinine, LDH, albumin, and arterial partial pressure of oxygen and carbon dioxide were recorded in the laboratory event table. In addition, the Sequential Organ Failure Assessment (SOFA) score and Simplified Acute Physiology Score II (SAPS II) were collected within 24 h of admission to the ICU. All comorbidities were identified according to the ICD-9 code records [10,12,14].

### 2.4 Study outcome

The primary outcome of our investigation was the incidence of in-hospital mortality. We established the start date for our follow-up as the day of the patient’s admission. We then gathered information regarding the date of death through the US government’s Social Security Death Index records. It is worth noting that the date of death was required to be prior to or on the date of hospital discharge to be considered for analysis [14].

### 2.5 Statistical analysis

In this study, we provided a comprehensive summary of the baseline patient data distribution across various outcome groups. Categorical variables were expressed as counts and percentages, while continuous variables were presented as mean values accompanied by standard deviations. To evaluate disparities in continuous variables, appropriate statistical tests such as analysis of variance or rank-sum tests were employed. Furthermore, for the comparison of categorical variables among different outcome groups, either chi-square tests or Fisher’s exact tests were utilized [15].

To investigate the potential independent association between the lymphocyte-to-absolute-neutrophil ratio (LAR) and in-hospital mortality, we developed multivariate logistic regression models employing a forward selection modeling approach. In the logistic regression model, the LAR ratio was included as a continuous variable, enabling us to examine its impact on the outcome. To present our findings, odds ratios (ORs) along with their corresponding 95% confidence intervals were utilized, offering a comprehensive representation of the results [10,15]. For the study outcome, we structured three multivariate models on the basis of LAR ratio groups. In model 1, we adjusted for covariates including age, sex, ethnicity, and LAR ratio. In model 2, we further adjusted model 1 plus history of diabetes, hypertension, history of liver disease, history of renal disease, and stroke [10]. Then, we continued adjusting for covariates including WBCs, platelets, hemoglobin, creatinine, BUN, sodium, SAPS-II, SOFA, SBP, DBP, HR, length of hospital stay, renal replacement therapy, use of vasopressin, and use of ventilator in model 3 [10]. To investigate the potential presence of a nonlinear association between in-hospital mortality and the LAR, we employed a generalized additive model to perform smooth curve fitting [16]. The resulting analysis provided a *P* value for trend, which was included in our study. To assess the discriminatory capacity of the LAR in distinguishing between patients who survived and those who did not, we employed receiver operating characteristic (ROC) curve analysis. All statistical computations were carried out using R software (version 4.1.1). Statistical significance was defined as a two-tailed *P* value less than 0.05 in accordance with established convention.

## 3 Results

### 3.1 Baseline characteristics of study population

The study included 2,177 patients after screening all patients in the database. Table 1 presents the demographic information and laboratory indexes of the final enrolled patients in our study. The mean age of the enrolled patients was 69.88 years for survivors and 71.95 years for nonsurvivors. Men made up the vast majority of the death group (63%). A total of 508 patients died in the hospital, and the mortality rate was 23.3%. In the survivor group, the mean LAR ratio was 13.44, while in the death group, the value was 17.38. Nonsurvivors had a longer length of ICU stay (7.76 vs 10.04, respectively; *P* = 0.001). Moreover, nonsurvivors had significantly higher SOFA and SAPSII scores than survivors (8.37 vs 6.48, *P* < 0.001; 52.5 vs 44.48, *P* < 0.001, respectively). In addition, survivors had lower Elixhauser scores, were less likely to require mechanical ventilation or the use of vasopressors, and were less likely to have renal disease and liver disease and diabetes. There were also significant differences in WBC count, platelet count, BUN, and creatinine between survivors and nonsurvivors (Table 1).

**Table 1:** Baseline characteristics of patients with acute heart failure, by survival status

	Survivors	Non-survivors	*P*-value
**Clinical parameters,** ***n*** **(%)**	1,669	508	
Age, years	69.88 (14.27)	71.95 (13.30)	0.004
Male, *n* (%)	941 (56.4)	320 (63.0)	0.01
**Ethnicity,** ***n*** **(%)**			<0.001
White	1,116 (66.9)	386 (76.0)	
Black	311 (18.6)	51 (10.0)	
Other	242 (14.5)	71 (14.0)	
Length of ICU stay, days	7.76 (8.22)	10.04 (9.57)	0.001
HR, beats/minute	84.59 (15.47)	85.41 (16.30)	0.301
SBP, mmHg	117.55 (17.83)	111.73 (16.69)	<0.001
DBP, mmHg	57.91 (10.95)	54.71 (10.45)	< 0.001
Vasopressin use, *n* (%)	110 (6.6)	147 (28.9)	<0.001
Ventilator use, *n* (%)	379 (22.7)	164 (32.3)	<0.001
Renal replacement therapy, *n* (%)	155 (9.3)	135 (26.6)	<0.001
**Laboratory parameters**			
WBC count, 10^9^/L	12.71 (9.69)	14.55 (10.65)	<0.001
Platelet count, 10^9^/L	218.89 (124.03)	201.75 (122.60)	0.006
Hemoglobin, g/dL	9.89 (1.88)	9.85 (1.88)	0.668
BUN, mg/dL	22.6(11.0–27.0)	45.5(26.0–56.0)	<0.001
Albumin, g/L	30.9 (0.62)	29.2 (0.69)	<0.001
Lactate dehydrogenase, U/L	498.72 ± 181.09	602.36 ± 349.33	0.001
Creatinine, mg/dL	1.3(0.9)	2.2(1.2)	0.001
Sodium, mmol/L	138.29 (5.75)	138.11 (5.87)	0.526
Glucose	151.2(102)	150(103.4)	0.965
PO_2_, mmHg	141.89 (69.39)	134.34 (68.30)	0.059
PCO_2_, mmHg	41.94 (11.13)	41.38 (11.82)	0.388
LDH/ALB	13.44 ± 8.68	17.38 ± 11.21	<0.001
**Scoring systems**			
SOFA	6.48 (2.97)	8.37 (3.44)	<0.001
SAPSII	44.48 (12.27)	52.50 (13.27)	<0.001
**Comorbidities**			
Hypertension, *n* (%)	1,354 (81.1)	384 (75.6)	0.008
Diabetes, *n* (%)	850 (50.9)	229 (45.1)	0.024
Renal disease, *n* (%)	227 (91.2%)	96 (18.9%)	0.003
Liver disease, *n* (%)	225 (13.5)	123 (24.2)	<0.001
Stroke, *n* (%)	109 (6.5)	50 (9.8)	0.016
Elixhauser comorbidity score	13.38 (7.06)	15.93 (7.71)	<0.001

### 3.2 Association between LAR and study outcome

In the multivariate logistic regression analysis, we developed three models to examine the relationship between the in-hospital death rate and the LAR. The adjusted variables in each model are shown in Table 2 and in Section 2. In models 1 and 2, the LAR was significantly associated with the rate of death (OR = 1.08, *P* < 0.001; OR = 1.09, *P* < 0.001, respectively). In model 3, after the adjustment of variables, the LAR was still significantly associated with in-hospital mortality (OR = 1.09, *P* < 0.001). We also identified the SAPSII score (OR = 1.11, *P* = 0.01), Elixhauser score (OR = 1.12, *P* = 0.003), vasopressin use (OR = 9.64, *P* < 0.001), and APACHE-II score (OR = 1.14, *P* = 0.006) as predictors of in-hospital death.

**Table 2:** ORs (95% CIs) for in-hospital mortality across groups of LDH/ALB ratio

Models	OR (95% confidence interval)	*P*-value
Model 1	1.08 (1.01–1.42)	<0.001
Model 2	1.09 (1.01–1.67)	<0.001
Model 3	1.09 (1.02–1.82)	<0.001

Model 1: Adjusted for age, sex, and ethnicity.

Model 2: Adjusted for model 1 plus history of diabetes, hypertension, history liver disease, history of renal disease, and stroke.

Model 3: Adjusted for model 2 plus WBCs, platelets, hemoglobin, creatinine, BUN, sodium, SAPS-II, SOFA, SBP, DBP, HR, length of hospital stay, renal replacement therapy, use of vasopressin, and use of ventilator.

**Figure 1:**
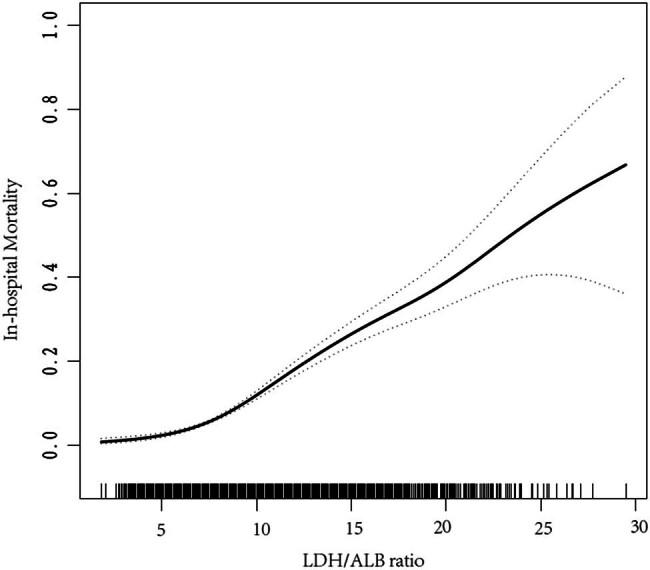
Relationship between LAR score and in-hospital mortality. The dotted lines on both sides represents 95% confidence interval.

To further elucidate the potential linear association between in-hospital mortality and the LAR, we proceeded to perform a smooth curve fitting analysis using R software based on model 3 (Figure 1). Remarkably, the *P* value for the trend of LAR in our study was found to be less than 0.001, indicating a significant relationship. By examining the curve, a distinct nearly linear relationship between in-hospital mortality and LAR becomes evident.

**Figure 2:**
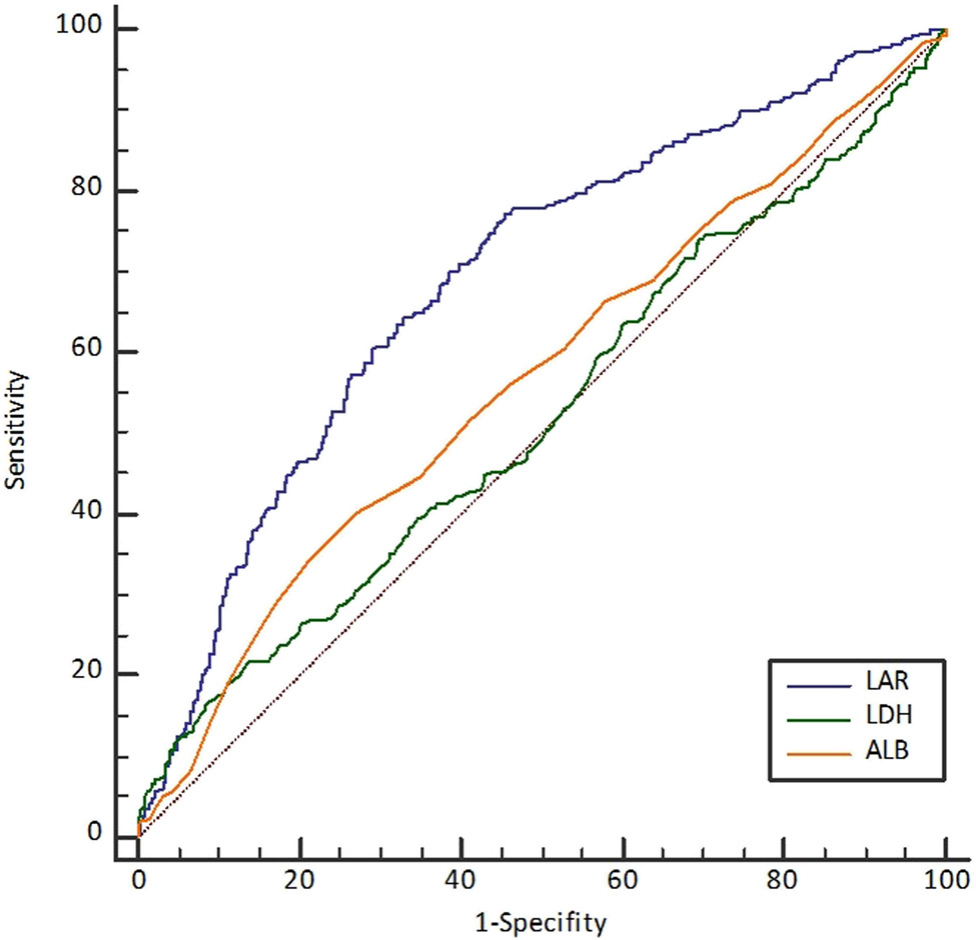
Performance in predicting in-hospital mortality using LAR.

We employed ROC analysis to assess the discriminative ability of the LAR in identifying patients at high risk of death. The area under the curve was calculated to be 0.75 (95% CI: 0.61–0.88, *P* < 0.001), indicating a favorable discriminatory performance (Figure 2). Notably, the LAR cutoff value with the highest sensitivity was determined to be 14.5, yielding a sensitivity of 91.8%, specificity of 65.6%, positive likelihood ratio of 2.75, and negative likelihood ratio of 0.23 in the detection of patients at elevated risk of mortality.

## 4 Discussion

The present study aimed to investigate the association between the LAR ratio and in-hospital mortality in patients with AHF. Our findings demonstrate that the LAR ratio is an independent risk factor for in-hospital mortality, along with advanced age, organ failure, and admission to the ICU. Notably, this study is the first to examine the prognostic effect of the LAR ratio in patients with AHF. Prior research has established the independent impact of the LAR ratio on mortality in critical infections requiring intensive care and pneumonia related to stroke in lower respiratory tract infections [17,18]. Thus, our study provides further evidence for the prognostic value of the LAR ratio in AHF and underscores the importance of monitoring this biomarker in clinical practice.

Numerous factors have been documented to exhibit correlation with the mortality rate of individuals diagnosed with HF. These include sex, age, body mass index, smoking status, left ventricular ejection fraction, New York Heart Association classification, presence of diabetes mellitus and chronic obstructive pulmonary disease, low SBP, serum creatinine levels, and non-utilization of beta-blockers and angiotensin-converting enzyme inhibitors/angiotensin II receptor blockers [4,19,20]. In our study higher LAR ratio was associated with higher in-hospital mortality.

Elevated levels of LDH have emerged as a compelling biomarker associated with unfavorable outcomes in patients with prior viral infections. Notably, COVID-19 patients have exhibited increased serum LDH levels, particularly among those with severe illness. Of significant interest, high initial LDH levels were found to be significantly correlated with a heightened risk of developing acute respiratory distress syndrome (ARDS) (HR: 1.61, 95% CI: 1.44–1.79) and mortality (HR: 1.30, 95% CI: 1.11–1.52) [5]. These findings underscore the potential clinical utility of monitoring LDH levels in COVID-19 patients and other viral infections, to facilitate early risk stratification and prompt interventions to improve patient outcomes [21].

Albumin is a multifunctional protein that plays a vital role in maintaining various physiological processes in the body. These functions include regulating osmotic pressure, vascular permeability, acid–base balance, and serving as anti-inflammatory and antioxidant molecules [22]. Previous studies have suggested that low levels of albumin (<3.5 g/dL) have been associated with increased 30-day mortality rates in the general hospitalized patient population [10]. A comprehensive meta-analysis of 90 studies also revealed that hypoalbuminemia is linked to prolonged ICU and hospital stays, as well as higher morbidity and mortality rates. Furthermore, albumin levels have been found to be decreased in patients with severe COVID-19, and such hypoalbuminemia has been demonstrated to be associated with increased mortality [5]. These findings highlight the crucial role of albumin as a prognostic biomarker in various clinical settings and underscore its potential as a therapeutic target in disease management.

Hoeboer et al. demonstrated the potential utility of LDH as a prognostic biomarker for predicting 28-day mortality in patients with ARDS [23] and for monitoring the severity and course of the disease. The present study found that albumin levels significantly affect mortality and were comparable between patient groups. In a related study, Lee et al. reported lower levels of albumin and higher levels of LDH and a higher LAR ratio in patients with lower respiratory tract infections [18]. Notably, the LAR ratio was identified as an independent factor associated with mortality in this study, underscoring its potential as a valuable prognostic biomarker.

In recent years, there has been some progress in understanding the relationship between chronic diseases and LDH and albumin. LDH, as an important intracellular catalyst, plays a crucial role in lactate metabolism, while albumin is one of the main proteins in plasma responsible for maintaining plasma osmotic pressure and carrying substances. Furthermore, chronic diseases can also affect albumin. Albumin is one of the most abundant proteins in plasma, and its synthesis and stability are regulated by many factors [14]. Chronic diseases such as diabetes and liver disease can lead to a decrease in albumin synthesis or an increase in degradation, resulting in a decrease in plasma albumin concentration. This phenomenon further leads to a decrease in plasma osmotic pressure and the occurrence of related issues such as edema. Chronic diseases such as liver disease and chronic renal failure all have unknown impact on LDH and albumin. In order to minimize such interference, we constructed multiple sets of regression models to evaluate the predictive role of the LDH/ALB ratio on mortality. The results showed that even after adjusting for certain chronic diseases, the LDH/ALB ratio remained an independent predictor of in-hospital mortality.This study has certain limitations that warrant acknowledgement. First, while we accounted for the primary confounding variables, there is a possibility of residual confounding variables that could influence our findings. Second, the scope of organ dysfunction evaluation tests utilized in this study was limited, which could impact the statistical power of the study. Additionally, the authors suggest that conducting similar investigations in diverse ethnic subgroups is crucial to establish the accuracy and dependability of our results.

## 5 Conclusions

To summarize, identifying factors that predict mortality is of paramount importance in patients with AHF, given the considerably elevated risks of both mortality and morbidity. Our investigation has established that a heightened LAR ratio is an independent risk factor associated with increased in-hospital mortality rates in patients with AHF.
